# Delivery of Long Non-coding RNA NEAT1 by Peripheral Blood Monouclear Cells-Derived Exosomes Promotes the Occurrence of Rheumatoid Arthritis via the MicroRNA-23a/MDM2/SIRT6 Axis

**DOI:** 10.3389/fcell.2020.551681

**Published:** 2020-09-11

**Authors:** Yujun Rao, Yuxuan Fang, Wei Tan, Dan Liu, Yubin Pang, Xia Wu, Chunwang Zhang, Guoqing Li

**Affiliations:** ^1^Department of Rheumatology and Immunology, Affiliated Hospital of Yangzhou University, Yangzhou University, Yangzhou, China; ^2^Department of Pathology, Clinical Medical College, Yangzhou University, Yangzhou, China

**Keywords:** rheumatoid arthritis, long non-coding RNA NEAT1, peripheral blood monouclear cells, exosomes, microRNA-23a, MDM2, SIRT6

## Abstract

Emerging evidence has pointed out the importance of long non-coding RNAs (lncRNAs) in multiple diseases, the knowledge of rheumatoid arthritis (RA)-associated lncRNAs remains limited. In this present study, we aimed to elucidate the mechanism of lncRNA nuclear paraspeckle assembly transcript 1 (NEAT1) from peripheral blood monouclear cell (PBMC)-derived exosomes (exos) on RA development by modulating the microRNA-23a (miR-23a)/murine double minute-2 (MDM2)/Sirtuin 6 (SIRT6) axis. RA was modeled *in vivo* by collagen induction in mice and *in vitro* by exposing fibroblast-like synoviocytes (FLSs) to lipopolysaccharide. Exos were isolated from human or mouse PBMCs, which were then were co-cultured with FLSs. Based on gain- and loss-of-function experiments, the cell proliferation and secretion of inflammatory factors were measured. LncRNA NEAT1 was found to be highly expressed in RA, and PBMCs-derived exos contributed to RA development by delivering lncRNA NEAT1. In lipopolysaccharide-induced FLSs, miR-23a inhibited the expression of MDM2, and overexpression of MDM2 partially rescued the inhibitory effect of miR-23a on FLS proliferation and inflammatory response. Mechanistically, MDM2 ubiquitination degraded SIRT6 in RA. LncRNA NEAT1 shuttled by PBMC-derived exos promoted FLS proliferation and inflammation through regulating the MDM2/SIRT6 axis. Furthermore, *in vivo* experiments suggested that downregulated lncRNA NEAT1 shuttled by PBMC-derived exos or upregulated miR-23a impeded RA deterioration in mice. This study highlights that lncRNA NEAT1 shuttled by PBMC-derived exos contributes to RA development with the involvement of the miR-23a/MDM2/SIRT6 axis.

## Introduction

Rheumatoid arthritis (RA) is a systemic autoimmune disease featured by chronic inflammation, persistent synovitis, and destruction of synovial joints (Scott et al., 2010). In addition to genetic and familial risk factors, there are multiple environmental, dietary as well as lifestyle factors have been demonstrated to be associated with risk of RA (Deane et al., 2017; Calabresi et al., 2018). Inflammatory cytokines, such as interleukin (IL)-6, IL-1β, and tumor necrosis factor (TNF)-α, have been revealed to play a role in the pathogenesis of RA (Harrison et al., 2008; Yamanaka, 2015; Solus et al., 2015). At present, the treatments for RA mainly focus on the immune system, inflammatory signaling pathways, and mediators (e.g., cytokines, kinases, proteases, and adhesion molecules implicated in the destructive process of the joints) (Oliveira and Fierro, 2018). Despite advances in these treatment approaches, the majority of patients with RA remain unremitted, hence, it is urgent to elucidate the molecular mechanism of RA for the purpose of identifying the potential therapeutic targets for RA (Liang et al., 2019).

Exosomes (exos), a class of nanoparticles, are endogenously secreted by mammalian cells, and the clinical applications for exos remain a challenge due to their low scalability, unsuitable donors, and insufficient targeting ability (Qi et al., 2016). The exos of stressed peripheral blood monouclear cells (PBMCs) have been found to enhance wound healing and angiogenesis *in vitro* and *in vivo* (Beer et al., 2015). Emerging evidence has demonstrated that long non-coding RNAs (lncRNAs) play a role in immunological functions and autoimmunity, and the differential expression of lncRNAs has been found in some autoimmune diseases, including RA (Wu et al., 2015). LncRNA nuclear paraspeckle assembly transcript 1 (NEAT1), located on chromosome 11q13.1, is expressed widely in numerous tissues and cell types (Huang et al., 2018). A study has suggested a high expression level of ncRNAs Hotair and NEAT1 in PBMCs and serum exos of patients with RA, contributing to the migration of active macrophages (Song et al., 2015). Of note, serum levels of microRNAs (miRNAs) also present differential profiles in multiple autoimmune diseases, including RA (Yamada et al., 2014). It is known that lncRNA NEAT1 can inhibit the expression of miR-23a (Zhao et al., 2019). miR-23a, a member of the miR-23a-27a-24-2 cluster, has been reported to modulate cell motility, cellular activation, and immune cell infiltration (Wade et al., 2019). At the systemic level, a research has stated that the relative expression of miR-23a increased in psoriatic arthritis (PsA) compared to osteoarthritis (OA) (Wade et al., 2019).

More importantly, bioinformatics analysis prior to our investigation revealed murine double minute-2 (MDM2), implicated in the occurrence of RA (Zhang et al., 2016), as a downstream gene of miR-23a. MDM2 is an intracellular molecule with the presence of diverse biological functions, often detected in multiple malignancies (Yuan et al., 2011). It is known that MDM2 can inhibit the expression of Sirtuin 6 (SIRT6) through its E3 ubiquitinase function (Thirumurthi et al., 2014). As reported, SIRT6 can suppress the activation of nuclear factor kappa B (NF-κB) signaling pathway by removing histone 3 lysine 9 (H3K9) acetylation levels of NF-κB downstream target genes (Kawahara et al., 2009). A prior study has shown that SIRT6 controls the cigarette smoke-induced signaling in RA synovial fibroblasts (Engler et al., 2014). More importantly, fibroblast-like synoviocytes (FLSs), a main component of synovial hyperplasia, have been highlighted as a critical regulator in RA, providing a potential therapeutic target for treating RA (Ganesan and Rasool, 2017). In addition, miRNAs are significantly implicated in FLSs during the progression of RA (Hong et al., 2018). Based on these evidences, we speculated that lncRNA NEAT1 shuttled by PBMC-derived exos might influence RA development with the involvement of the miR-23a/MDM2/SIRT6 axis.

## Materials and Methods

### Ethics Statement

The study protocols were approved by the Ethic Committee of Affiliated Hospital of Yangzhou University (approval number: 2020-YKL03-004). All participants signed the written informed consents. All animal experiments were in compliance with the *Guide for the Care and Use of Laboratory Animal* by National Institutes of Health. Great efforts had been made to minimize the pain of animals.

### Establishment of a Mouse Model of RA

Thirty-two specific pathogen free healthy DBA/1J mice (6 weeks, 16–20 g) were purchased from the Animal Research Center of Affiliated Hospital of Yangzhou University and 24 of them were selected for establishing the RA mouse models according to a previous study (Duan and Li, 2018). Briefly, DBA/1J mice were injected with a mixture (100 μL) of 100 μg bovine type II collagen (2 mg/mL) and an equal volume of Freund’s adjuvant (5 mg/mL) *via* tail. After 21 days, an enhanced injection was performed using a mixture of 100 μg bovine type II collagen (2 mg/mL) and an equal volume of Freund’s adjuvant. Five weeks later, RA mice were randomly assigned into 3 groups (*n* = 8): RA group (successfully modeled RA mice), RA + human controls-isolated PBMCs-derived exos infected with lentivirus harboring negative control (NC) for short hairpin RNA (shRNA) (hC-PBMC-exo ^shNC^) group (RA mice were injected with exos from PBMCs of human controls that had been infected with lentivirus harboring sh-NC) and RA + hC-PBMC-exo ^shNEAT1^ group (RA mice were injected with exos from PBMCs of human controls that had been infected with lentivirus harboring sh-NEAT1). On the 36th, 38th, 40th, 42nd, and 44th days, exos [concentration of 10 × 10^8^ particles, dissolved in 50 μL phosphate buffered saline, (PBS)] were injected intravenously into RA mice (Wen et al., 2016; Riau et al., 2019). Healthy mice injected with the vehicle (*n* = 8) were considered as controls. As previously described (Brand et al., 2007), the severity of arthritis in each limb of the mouse was measured as follows, 0 point, no evidence of erythema and swelling; 1 point, erythema and mild swelling confined to the tarsals or ankle joint; 2 points, erythema and mild swelling from the ankle to the tarsals; 3 points, erythema and moderate swelling from the ankle to metatarsal joints; 4 points, erythema and severe swelling encompass the ankle, foot and digits, or ankylosis of the limb.

### Isolation and Culture of FLSs

According to a previous study (Franks et al., 2006), FLSs were isolated from normal and RA mice. Briefly, the synovium of the knee joint from unimmunized control mice was isolated and then detached with 1 mg/mL collagenase A (Roche Diagnostics, Basel, Switzerland) in serum-free Roswell Park Memorial Institute (RPMI) 1640 medium (Gibco by Life technologies, Grand Island, NY, United States) for 2 h at 37°C. The tissue digests were suspended and filtered through nylon mesh. Dissociated cells were washed 3 times in RPMI 1640 medium supplemented with 50 μM 2-mercaptoehtanol, 5 mM sodium pyruvate, and 10% heat-inactivated fetal calf serum (Gibco) and then cultured overnight. The non-adherent cells were removed. The confluent cells were trypsinized, divided into three parts, and re-plated. The passage was repeated 3–4 times before use. The stably growing FLSs were cultured in RPMI 1640 medium (Gibco) containing 10% fetal bovine serum (FBS) and 1% penicillin and streptomycin (P/S) (Gibco) at 37°C with 5% CO_2_. FLSs between passage 4 and 8 were used for further analysis. Using antibodies against fibroblast marker CD90 and macrophage marker CD14 (Abcam, Cambridge, United Kingdom), pure FLSs (> 90% CD90^+^/ < 1% CD14^+^) were identified by flow cytometry. FLSs in logarithmic growth phase were used for cell transfection or lentivirus infection. The plasmids or lentivirus used in our study were provided by Shanghai GenePharma Co., Ltd. (Shanghai, China). FLSs were transfected with miR-23a mimic/mimic-NC according to lipo3000 (Sigma-Aldrich, St. Louis, United States). When the cells in the logarithmic growth phase reached about 30% confluence, 2 × 10^6^ TU of the lentivirus harboring overexpressed (oe)-NC, oe-SIRT6, or oe-MDM2 (GenePharma, Shanghai, China) and 5 μg Poly-brene were supplemented into 1 mL medium without serum or antibacterial drug. After 48 h of incubation, 1 μg/mL puromycin was added to each well to select stalely transfected cells. MG132 (5 μM) treatment was performed for 24 h according to the literature (Migita et al., 2001) or at the same time, an RA model of FLSs at the logarithmic phase induced by 24-h cultured in medium containing 10 μg/mL lipopolysaccharide (LPS) was established according to the literature (Zhang H. J. et al., 2017).

### Enzyme-Linked Immunosorbent Assay (ELISA)

According to the instructions, ELISA was used to detect the secretion of inflammatory factors in the supernatant of FLSs and mouse serum: IL-6 (ab100712), IL-1β (ab197742), and TNF-α (ab208348) (Abcam).

### Isolation, Acquisition, and Transfection of PBMCs

Peripheral blood was collected from patients with RA (*n* = 5) and healthy volunteers (*n* = 5) of matched age who underwent knee replacement surgery in Affiliated Hospital of Yangzhou University. Peripheral blood was also collected from normal and RA mice. According to the literature (Mocharla et al., 2013), PBMCs were isolated and obtained. In brief, PBMCs were plated at a density of 0.5–1 × 10^6^ cells/mL with a total volume of 0.5–1 mL in a 24-well plate, using RPMI-1640 medium (Gibco) supplemented with 10% FBS + 1% P/S (Gibco), and further incubated at 37°C with 5% CO_2_. PBMCs in logarithmic growth phase were infected with lentiviruses harboring sh-NC and sh-NEAT1 (GenePharma) by adding 2 × 10^6^ TU corresponding lentivirus and 5 μg Poly-brene into 1 mL medium in the absence of serum or antibacterial drug. Forty-eight hours later, 1 μg/mL puromycin was added to each well to screen the stably transfected cells.

### Exo Isolation and Identification

When the PBMCs reached 80–90% confluence, the complete medium was removed, and the cell culture medium (30 mL) was collected form each cell line. Exos were isolated from culture supernatants through differential centrifugation, at 300 × g for 10 min, at 2,000 × g for 10 min, at 10,000 × g for 30 min and at 100,000 × g for 70 min, followed by one PBS wash and purification through centrifugation at 100,000 × g for 70 min. Next, the pellet after centrifugation was resuspended in PBS and stored at −80°C for further use.

All qualified blood samples (from normal human, RA patient, normal mouse, and RA mouse) were coagulated at room temperature and centrifuged at 3000 × g for 10 min. The cell debris was removed by centrifuging the serum at 3000 × g (4°C) for 10 min, and at 10,000 × g (4°C) for 30 min. Next, 1 mL supernatant was then ultracentrifuged (Class H, R, and S Preparative Ultracentrifuges, Type 50.4 Ti Rotor; Beckman Coulter, Brea, CA, United States) at 100,000 × g (4°C) for 2 h to pellet the particles. The particles were washed with PBS, and then filtered through a 0.22 μm filter, and then subjected to a second ultracentrifugation at 100,000 × g (4°C) for 2 h to precipitate exos. Exo particles were resuspended in 100 μL PBS.

Identification of exos: the sample was adsorbed onto a carbon-coated nickel grid and stained with 2% methylamine tungstate for 5 min. The stain was then removed and rinsed twice with distilled water, followed by sample drying. Next, the sample was examined at an 80 kV acceleration voltage in an electron microscope (JEM-1230, Nihon Denshi, Tokyo, Japan) (Zhang X. et al., 2017). Particle size analysis for exos was conducted with the nanoparticle tracking analysis (NS300, Malvern Instruments Ltd, Worcestershire, United Kingdom) (Fang et al., 2018). The suspension of exos was concentrated to determine the protein content by bicinchoninic acid kit (23227, Thermo Fisher Scientific, Waltham, MA, United States). Sodium dodecyl sulfate polyacrylamide gel electrophoresis (SDS-PAGE) was prepared for protein denaturation and electrophoresis, followed by the membrane transferring. The expression of exo specific marker proteins, tumor susceptibility gene 101 (TSG101), CD63 and CD81, were detected (Li et al., 2013).

### Labeling and Tracking of Exos

Based on the manufacturer’s instructions, the 20 μg exos were labeled with PKH26 Red Fluorescent membrane linker dye (Sigma-Aldrich). After that, the labeled exo particles were then resuspended and supplemented to the unstained FLSs for uptake of exos. After incubation at 37°C for 12 h, the uptake of exos by FLSs were viewed by a confocal microscope (Zeiss Meta 510, Thornwood, NY, United States).

### Co-culture of FLSs and PBMC-Derived Exos

Fluorescently labeled exos were incubated with FLSs that had been seeded in a 24-well plate with 50–60% confluence for 48 h before subsequent experiments. The co-culture systems were grouped into: control (FLSs treated with PBS), hC-exo (co-culture of PBMC-derived exos from human controls with FLSs), hRA-exo (co-culture of PBMC-derived exos from RA patients with FLSs), mC-exo (co-culture of PBMC-derived exos from normal mouse with FLSs), mRA-Exo (co-culture of PBMC-derived exos from RA mouse with FLSs), hRA-exo + mimic-NC (co-culture of PBMC-derived exos from RA patients with FLSs that had been transfected with mimic-NC), hRA-exo + miR-23a mimic (co-culture of PBMC-derived exos from RA patients with FLSs that had been transfected with miR-23a mimic), hC-PBMC-exo (co-culture of exos secreted from PBMCs of human controls with FLSs), hRA-PBMC-exo (co-culture of exos secreted from PBMCs of RA patients with FLSs), hRA-PBMC-exo^shNC^ (co-culture of exos secreted from PBMCs of RA patients that had been infected with sh-NC with FLSs), hRA-PBMC-exo ^shNEAT1^ (co-culture of exos secreted from PBMCs of RA patients that had been infected with shNEAT1 with FLSs), hRA-PBMC-exo^shNEAT1^ + mimic-NC (co-culture of exos secreted from PBMCs of RA patients that had been infected with shNEAT1 with FLSs that had been transfected with mimic-NC), hRA-PBMC-exo ^shNEAT1^ + miR-23a mimic (co-culture of exos secreted from PBMCs of RA patients that had been infected with shNEAT1 with FLSs that had been transfected with miR-23a mimic), hRA-PBMC exo ^shNEAT1^ + oe-NC (co-culture of exos secreted from PBMCs of RA patients that had been infected with shNEAT1 with FLSs that had been transfected with oe-NC), hRA-PBMC-exo ^shNEAT1^ + oe-MDM2 (co-culture of exos secreted from PBMCs of RA patients that had been infected with shNEAT1 with FLSs that had been transfected with oe-MDM2) and hRA-PBMC-exo ^shNEAT1^ + oe-SIRT6 (co-culture of exos secreted from PBMCs of RA patients that had been infected with shNEAT1 with FLSs that had been transfected with oe-SIRT6) groups.

### Reverse Transcription Quantitative Polymerase Chain Reaction (RT-qPCR)

TRIzol reagent (Invitrogen, Carlsbad, CA, United States) was used to extract total RNA based upon the manufacturer’s instructions. RT was carried out using a PrimeScript RT reagent Kit (Promega, Madison, WI, United States). Subsequently, gene expression was quantified with the application of SYBR Green Master Mix (Life Technologies, Carlsbad, CA, United States). For miRNA detection, the cDNA was obtained by RT using the Mir-XTM miRNA First Strand Synthesis Kit (Takara, Dalian, Liaoning, China). Quantification of miR-23a was checked with Mir-XTM miRNA RT-qPCR TB Green^TM^ Kit (Takara). Primer sequences are listed in Table 1. The 2^–ΔΔCt^ method was used for calculating the expression of genes. U6 was a loading control of miR-23a, and glyceraldehyde phosphate dehydrogenase (GAPDH), an internal reference of other genes. In addition, before isolating extracellular RNA (exos), 25 fmol of C.elegans cel-miR-39 (Ribobio, Guangzhou, China) was supplemented to each sample. cel-miR-39 was used to normalize the content in exos.

**TABLE 1:** Primer sequence for RT-Qpcr.

**Gene**	**Forward (5′–3′)**	**Reverse (5′–3′)**
h-NEAT1	GCCTTCTTGTGCGTTTCTCG	TCCCAGCGTTTAGCACAACA
m-NEAT1	TGGCTAGCTCAGGGCTTCAG	TCTCCTTGCCAAGCTTCCTTC3′
hsa-miR-23a-3p	GCGATCACATTGCCAGGG	CAGTGCGTGTCGTGGAGT
mmu-miR-23a-3p	ATCACATTGCCAGGGATT	CTCAACTGGTGTCGTGGA
h-U6	CTCGCTTCGGCAGCACA	AACGCTTCACGAATTTGCGT
m-U6	GCATGACGTCTGCTTTGGA	CCACAATCATTCTGCCATCA
h-GAPDH	CGGAGTCAACGGATTTGGTCGTAT	AGCCTTCTCCATGGTGGTGAAGAC
m-GAPDH	TGGCAAAGTGGAGATTGTTG	GTCTTCTGGGTGGCAGTGAT

*h, human; m, mouse; NEAT1, nuclear paraspeckle assembly transcript 1; miR, microRNA; GAPDH, glyceraldehyde-3-phosphate dehydrogenase; RT-qPCR, reverse transcription quantitative polymerase chain reaction.*

### Western Blot Analysis

The total protein was extracted from tissues and cells by radioimmunoprecipitation assay buffer containing phenylmethyl sulfonylfluoride (P0013C, Beyotime Co., Ltd., Shanghai, China) and incubated on ice for 30 min. The supernatant was collected through centrifugation at 4°C for 10 min at 8,000 × g. The concentration of total proteins was measured by bicinchoninic acid kits (BCA1 AND B9643, Sigma-Aldrich). The extracted proteins (50 μg) were dissolved in 2 × SDS loading buffer solution, boiled at 100°C for 5 min, separated by 10% SDS-PAGE, and then transferred onto the polyvinylidene difluoride membranes in a wet manner. Next, the membranes were blocked with 5% bovine serum albumin for 1 h, and then incubated with primary antibodies against phosphorylated p65 (p-p65) (ab86299, 1 : 2000), GAPDH (ab181602, 1 : 10000), TSG101 (ab125011, 1 : 1000), CD63 (ab216130, 1 : 1000), CD81 (ab109201, 1 : 1000), Calnexin (ab10286, 1 : 1000), SIRT6 (ab191385, 1 : 2000), and MDM2 (ab38618, 1: 1000) and then re-probed with the horseradish peroxidase-conjugated goat anti-rabbit secondary antibody (ab205718, 1 : 2000) (all from Abcam). Following development by chemiluminescence reagent and Gel imaging, Bio-Rad image analysis system (Bio-Rad, Hercules, CA, United States) was used to take pictures and results were analyzed with Quantity One v4.6.2 software. The relative protein content was expressed by the gray value of the corresponding protein band/the gray value of GAPDH.

### Immunoprecipitation Assay

For co-immunoprecipitation of SIRT6 with MDM2 and ubiquitin, 50–75 μg of protein lysate was immunoprecipitated with SIRT6 antibody (ab191385, 1 : 40, Abcam) overnight at 4°C. Protein A/G agarose (10 mL/sample, Pierce, Dallas, TX, United States) were added into the tubes and rotated for 2 h at 4°C. Beads were precipitated through centrifugation at 1000 × g for 15 s and washed 3 times with cold lysis buffer. Then, 1.5 × SDS loading buffer was used to resuspend the pellet, followed by incubation at 98°C for 6 min. The supernatants were harvested and used for Western blot analysis.

### Immunohistochemistry

Paraffin sections of mouse synovial tissues were dewaxed, hydrated without antigen retrieval, immersed in RNase containing 3% H_2_O_2_ for 10 min, blocked with 10% normal goat serum for 10 min and incubated with rabbit anti-Ki67 polyclonal antibody (ab15580, 1 : 200, Abcam) at 4°C overnight. Next, the biotin-labeled goat anti-rabbit immunoglobulin G secondary antibody (ab6721, 1: 1000, Abcam) was supplemented and incubated with sections for 10 min. Following addition of SP solution, diaminobenzidine was added for development, and hematoxylin was introduced for counterstaining. The neutral gum-mounted sections were observed and recorded under a microscope (Olympus, Tokyo, Japan). The yellowish-brown staining of the cytoplasm was defined as positive staining. Under light microscopy, five high-power fields were randomly selected and the average value was calculated. The total number of positive cells and the percentage of total cells were used to calculate the positive rates.

### Luciferase Activity Assay

A website (starbase)^1^ was used to predict whether miR-23a could target MDM2. The dual luciferase reporter gene assay was used to verify whether there was a binding site between miR-23a and MDM2. The pGL3-MDM2 wild type (Wt) and pGL3-MDM2 mutant type (Mut) were co-transfected with miR-23a mimic/mimic-NC and pRL-TK (internal reference plasmids expressing renilla luciferase) into HEK293 cells. After 24 h of transfection, the cells were lysed according to the steps of TransDetect Double-Luciferase Reporter Assay Kit (FR201-01, TransGen Biotech, Beijing, China) and the supernatant was collected. The dual-luciferase reporter assay system (E1910, Promega) was used to detect luciferase activity. The relative luciferase activity of firefly luciferase/renilla luciferase was used as the relative luciferase activity. Similarly, pGL3-NEAT1 Wt and pGL3-NEAT1 Mut were constructed, and then luciferase activity was detected in the same way for verification on binding between lncRNA NEAT1 and miR-23a.

### Cell Counting Kit-8 (CCK-8) Assay

The procedures were carried out based upon the CCK-8 kit (C0037, Beyotime, Shanghai, China), and 1 × 10^5^ cells were seeded into 96-well plates per well, followed by 24-h treatment. CCK-8 reagent (10 μL) was added to 100 μL complete medium, and the absorbance value at 450 nm wavelength was detected at different time points (0, 12, 24, and 48 h) by a microplate reader (Multiskan FC, 51119100, Thermo Fisher Scientific).

### Statistical Analysis

Analyses were performed with statistical software (SPSS 21.0; IBM Corp., Armonk, NY, United States). The measurement data were depicted as mean ± standard deviation. The two sets of data subjected to normal distribution and homogeneity of variance with an unpaired design were compared using unpaired *t*-test, while the data among multiple groups was analyzed using one-way analysis of variance (ANOVA), followed by Tukey’s *post hoc* test. Cell viability at different time points was compared using two-way ANOVA, followed by Bonferroni *post hoc* test. *p* < 0.05 indicated that the difference was statistically significant.

## Results

### Highly-Expressed LncRNA NEAT1 in PBMC-Derived Exos Promotes RA Development

**FIGURE 1:**
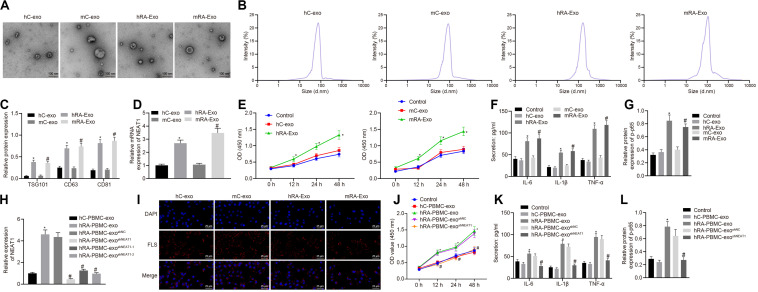
Highly expressed lncRNA NEAT1 in PBMC-derived exos contributes to RA. **(A)** The morphology of exos in human or mouse blood observed by a transmission electron microscope (scale bar = 100 nm). **(B)** Nanoparticle tracking analysis used to analyze the size distribution of the exos. **(C)** Western blot analysis used to detect the Exo marker proteins TSG101, CD63, and CD81. **(D)** Detection of lncRNA NEAT1 expression in PBMC-derived exos from human or mouse blood by RT-qPCR. **(E)** Detection of viability of FLSs by CCK-8 assay. **(F)**. Detection of the secretion of inflammatory factors in the culture medium of FLSs by ELISA. **(G)** Detection of phosphorylation of p65 normalized to GAPDH in FLSs by Western blot analysis. **(H)** Detection of lncRNA NEAT1 expression in FLSs by RT-qPCR. **(I)**. Detection of endocytosis of exos by FLSs using immunofluorescence assay (400×). **(J)** Detection of viability of FLSs by CCK-8 assay. **(K)** Detection of the secretion of inflammatory factors in the culture medium of FLSs by ELISA. **(L)**. Detection of phosphorylation of p65 normalized to GAPDH in FLSs by Western blot analysis. **(C–G)** *p < 0.05 vs. the hC-exo group; ^#^*p* < 0.05 vs. the mC-exo group. **(H–L)** *p < 0.05 vs. hC-PBMC-exo group; ^#^*p* < 0.05 vs. the hRA-PBMC-exo^*sh–NC*^ group. Normal FLSs served as the control group. The measurement data were depicted as mean ± SD. The data among multiple groups was analyzed using one-way ANOVA, followed by Tukey’s *post hoc* test. Cell viability at different time points was compared using two-way ANOVA, followed by Bonferroni *post hoc* test. Each experiment was run in triplicate.

In order to confirm the expression of lncRNA NEAT1 in PBMC-derived exos of patients with RA and their roles in RA, we firstly collected the peripheral blood from normal people and patients with RA, and then isolated the exos from the peripheral blood. Observed by a transmission electron microscope, we found that the shape of exos was solid and dense, with a typical double-layered membrane structure, which was disk or cup-shaped, with an average diameter of about 90 nm (Figures 1A,B). Western blot analysis was used to detect the Exo marker proteins TSG101, CD63, and CD81. Compared with the hC-exo group, the Exo marker proteins TSG101, CD63, and CD81 were significantly increased in the hRA-exo group (Figure 1C), indicating successful extraction of hC-exo and hRA-exo with more exos detected in hRA. Meanwhile, contrast to the hC-exo group, the expression of lncRNA NEAT1 in the hRA-exo group was significantly increased (Figure 1D). Similarly, the adjuvant-induced RA mouse models showed similar results (Figures 1A–D). Of note, the NF-κB signaling pathway has been reported to play an important role in RA (Duan and Li, 2018) while p-p65 is a common indicator to evaluate the activity of the NF-κB signaling pathway (Hua et al., 2013; Li et al., 2017). To further investigate the action of lncRNA NEAT1 on RA, FLSs were isolated from mice and co-cultured with exos derived from PBMCs, followed by cell viability assessment, secretion of inflammatory factors in the supernatants and p-p65 quantification. Compared with the hC-exo group and the mC-exo group, FLS viability and secretion of inflammatory factors (IL-6, IL-1β, and TNF-α) and phosphorylation of p65 were increased in the hRA-exo group and the mRA-exo group (Figures 1E–G). For exploration on the action of lncRNA NEAT1 in RA, PBMCs were isolated from patients with RA and healthy volunteers while lncRNA NEAT1 expression was silenced by lentivirus. NEAT1 encodes two transcript variants, i.e., NEAT1-1 and NEAT1-2, both of which share similar functions (Yu et al., 2017). To ensure the post-transcriptional silencing efficiency of shRNA, different shRNA sequences were designed based on two subtypes. RT-qPCR for lncRNA NEAT1 expression in PBMCs indicated that relative to the hC-PBMC-exo group, the expression of lncRNA NEAT1 elevated in the hRA-PBMC-exo group; in comparison to the hRA-PBMC-exo ^*shNC*^ group, the hRA-PBMC-exo ^*shNEAT1*^ group, the hRA-PBMC-exo^shNEAT1–1^ group and the hRA-PBMC-exo ^*shNEAT1–2*^ group presented decreased expression of lncRNA NEAT1, among which the hRA-PBMC-exo^shNEAT1^ group had the lowest lncRNA NEAT1 expression, which was therefore selected for subsequent experiments (Figure 1H). Next, PKH26-labeled PBMC-derived exos were then co-cultured with FLSs, and it was found that PBMC-derived exos could be up-taken by FLSs (Figure 1I). As for evaluation on FLS cellular functions after co-culture, it was found that compared with the hC-PBMC-exo group, the FLS viability and secretion of inflammatory factors (IL-6, IL-1β, and TNF-α) and phosphorylation of p65 in the hRA-PBMC-exo group were significantly increased; while vs. the hRA-PBMC-exo^shNC^ group, the hRA-PBMC-exo ^*shNEAT1*^ group showed the opposite trend (Figures 1J–L). These results suggest that lncRNA NEAT1 is highly expressed in PBMC-derived exos in contribution to RA development.

### miR-23a Partially Reverses the Effects of PBMC-Derived Exos on FLSs

**FIGURE 2:**
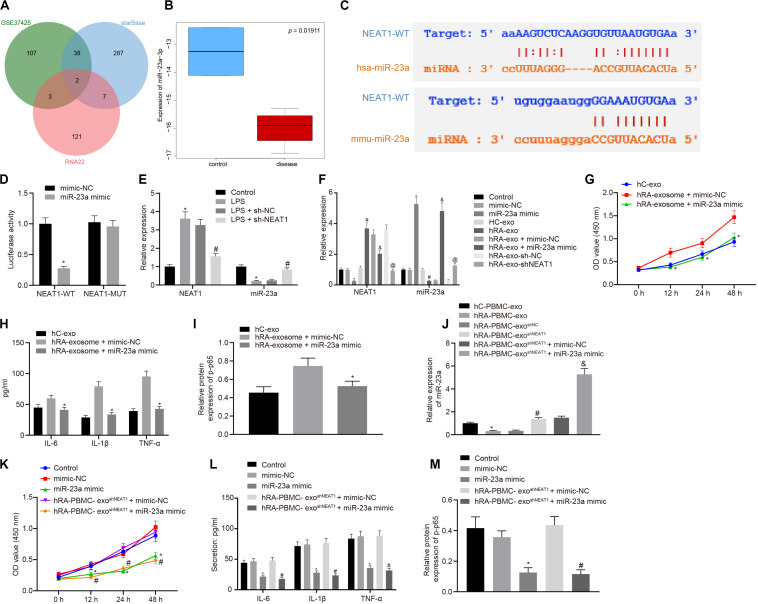
miR-23a partially reverses the effects of PBMC-derived exos on FLSs. **(A)** RNA22 and starBase databases predicted the Venn plots of miRNAs with significant differences in downstream miRNAs of lncRNA NEAT1 and GSE37425 microarray dataset. The intersection miRNAs were hsa-miR-339-5p and hsa-miR-23a-3p. **(B)** miR-23a expression pattern in GSE37425 microarray dataset. The blue box on the left represents the expression of normal samples, and the red box on the right represents the expression of RA samples. **(C)** starBase predicted the binding sites of miR-23a from human (top) and mouse (bottom) to lncRNA NEAT1. **(D)** Luciferase activity assay for the binding site between miR-23a and lncRNA NEAT1. *p < 0.05 vs. the mimic-NC group. **(E)** Detection of lncRNA NEAT1 and miR-23a expression in FLSs by RT-qPCR. *p < 0.05 vs. the control group; #*p* < 0.05 vs. the LPS + sh-NC group. **(F)** Detection of lncRNA NEAT1 and miR-23a expression in FLSs in each group by RT-qPCR. *p < 0.05 vs. the mimic-NC group; #*p* < 0.05 vs. the hC-exo group; &*p* < 0.05 vs. the hRA-exo + mimic-NC group; @*p* < 0.05 vs. the hRA-exo + sh-NC group. **(G)**. Detection of viability of FLSs by CCK-8 assay. **(H)** Detection of the secretion of inflammatory factors in the culture medium of FLSs by ELISA. **(I)** Detection of phosphorylation of p65 normalized to GAPDH in FLSs by Western blot analysis. **(G–I)** *p < 0.05 vs. the hRA-exo + mimic-NC group. **(J)** Detection of miR-23a expression in FLSs in each group by RT-qPCR. *p < 0.05 vs. the hC-PBMC-exo group; ^#^*p* < 0.05 vs. the hRA-PBMC-exo^*sh–NC*^ group; ^&^*p* < 0.05 vs. the hRA-PBMC-exo^*sh–NC*^ + mimic-NC group. **(K)** Detection of viability of FLSs by CCK-8 assay. **(L)** Detection of the secretion of inflammatory factors in the culture medium of FLSs by ELISA. **(M)** Detection of phosphorylation of p65 normalized to GAPDH in FLSs by Western blot analysis. **(K–M)** *p < 0.05 vs. the mimic-NC group; #*p* < 0.05 vs. the hRA-PBMC-exo^*sh–NC*^ + mimic-NC group. The measurement data were depicted as mean ± standard deviation. The two groups were compared by non-paired *t*-test. The data among multiple groups was analyzed using one-way ANOVA, followed by Tukey’s *post hoc* test. Cell viability at different time points was compared using two-way ANOVA, followed by Bonferroni *post hoc* test. Each experiment was run in triplicate.

Through the analysis of R language^2^, the RA-related miRNA microarray dataset GSE37425 from Gene Expression Omnibus (GEO) database^3^ showed that there were 150 differentially expressed miRNAs in RA. Furthermore, RNA22 (Folding energy < -29)^4^ and starBase (clipExpNum ≥ 10)^5^ were used to predict the downstream miRNAs of lncRNA NEAT1, revealing 133 and 334 miRNAs, respectively. The intersection of GSE37425 microarray dataset and downstream miRNAs resulted in two key miRNAs: hsa-miR-339-5p and hsa-miR-23a-3p (Figure 2A). It is known that lncRNA NEAT1 can inhibit the expression of miR-23a (Zhao et al., 2019). The boxplot of miR-23a expression data from GSE37425 microarray dataset showed that it was poorly expressed in RA (Figure 2B). The starBase also provided the probable binding sites of human and mouse lncRNA NEAT1 to miR-23a (Figure 2C). With the aim to probe into whether the role of lncRNA NEAT1 in RA is related to miR-23a, we firstly tested the binding site between miR-23a and lncRNA NEAT1 using luciferase activity assay. The results indicated that in HEK293T cells, miR-23a mimic inhibited the luciferase activity in the NEAT1-Wt group, but has no significant effect on the luciferase activity in the NEAT1-Mut group (Figure 2D). Next, LPS-treated FLSs were used to construct the RA cell model. The results showed that elevated lncRNA NEAT1 and declined miR-23a were found in the LPS-induced RA cell model. After knocking down lncRNA NEAT1, the expression of lncRNA NEAT1 decreased, while miR-23a expression increased (Figure 2E). The PBMC-derived exos were incubated with FLSs carrying miR-23a mimic, and we found increased miR-23a expression and reduced lncRNA NEAT1 expression. Compared with the hC-exo group, the expression of lncRNA NEAT1 increased and the expression of miR-23a decreased in the hRA-exo group; relative to the hRA-exo + mimic-NC group and the hRA-exo + sh-NC, increased miR-23a and reduced lncRNA NEAT1 were witnessed in the hRA-exo + miR-23a mimic group and the hRA-exo + shNEAT1 group (Figure 2F). These results suggest that lncRNA NEAT1 from RA PBMC-derived exos can reduce miR-23a expression in FLSs. vs. the hRA-exo + mimic-NC group, the cell viability and secretion of inflammatory factors (IL-6, IL-1β, and TNF-α) and phosphorylation of p65 were decreased in the hRA-exo + miR-23a mimic group (Figures 2G–I). The results suggest that overexpression of miR-23a can inhibit the promotion of FLS viability and inflammation by PBMC-derived exos in patients with RA.

The following steps were to detect the expression of miR-23a after FLS incubation of PBMC-derived exos in patients with RA and human controls. The findings indicated that miR-23a expression decreased in the hRA-PBMC-exo group in comparison to the hC-PBMC-exo; miR-23a expression was enhanced in the hRA-PBMC-exo^shNEAT1^ group and the hRA-PBMC-exo^shNEAT1^ + miR-23a mimic group in contrast to the hRA-PBMC-exo^shNC^ group and the hRA-PBMC-exo^shNEAT1^ + mimic-NC group (Figure 2J). The cell viability and secretion of inflammatory factors (IL-6, IL-1β, and TNF-α) and phosphorylation of p65 were decreased in the hRA-PBMC-exo^shNEAT1^ + miR-23a mimic group and the miR-23a mimic group relative to the hRA-PBMC-exo^shNEAT1^ + mimic-NC group and the mimic-NC group (Figures 2K–M).

### miR-23a Inhibits Inflammation in RA by Suppressing MDM2

**FIGURE 3:**
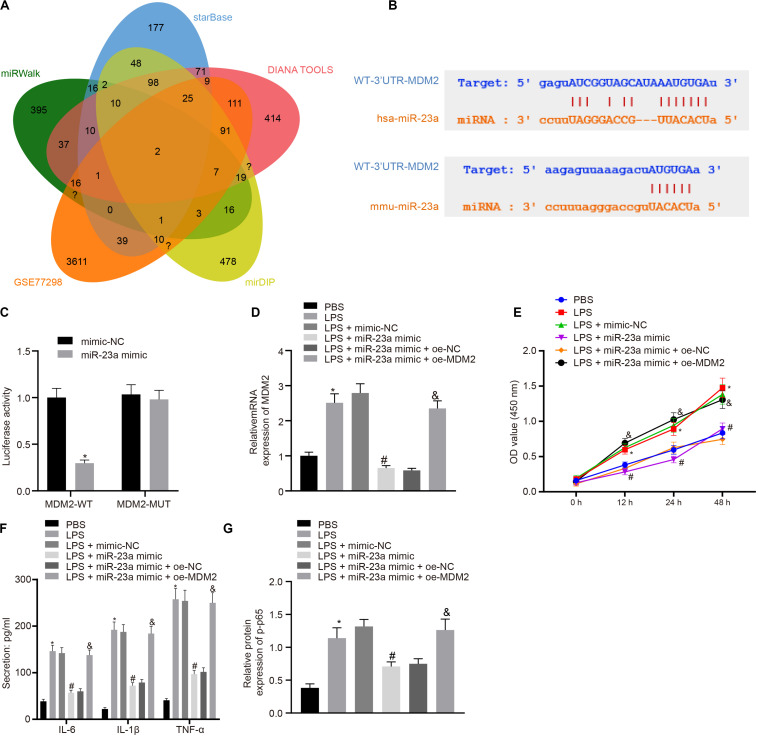
miR-23a inhibits MDM2 expression in RA. **(A)**. miRWalk, starBase, DIANA TOOLS and mirDIP databases predicted the Venn plots of genes with significant differences in the downstream gene of miR-23a and GSE77298 microarray dataset. The intersection gene were PDE7A and MDM2. **(B)** starBase predicted the binding sites of miR-23a from human (top) and mouse (bottom) to the target gene MDM2. **(C)** Luciferase activity assay validated the binding site between miR-23a and MDM2. *p < 0.05 vs. the mimic-NC group. **(D)** Detection of MDM2 expression in FLSs by RT-qPCR. **(E)** Detection of viability of FLSs by CCK-8 assay. **(F)** Detection of the secretion of inflammatory factors in the culture medium of FLSs by ELISA. **(G)** Detection of phosphorylation of p65 normalized to GAPDH in FLSs by Western blot analysis. **(D–G)**: *p < 0.05 vs. the PBS group; #*p* < 0.05 vs. the LPS + mimic-NC group; &*p* < 0.05 vs. the LPS + miR-23a + oe-NC group. The measurement data were depicted as mean ± standard deviation. The two groups were compared by non-paired *t-*test. The data among multiple groups was analyzed using one-way ANOVA, followed by Tukey’s *post hoc* test. Cell viability at different time points was compared using two-way ANOVA, followed by Bonferroni *post hoc* test. Each experiment was run in triplicate.

In order to find out the role of miR-23a in RA, we predicted the downstream genes of miR-23a through miRWalk (energy < −19, accessibility < 0.05, au > 0.6)^6^, starBase (clipExpNum > 10), DIANA TOOLS (miTG score > 0.75)^7^, and mirDIP (Integrated Score > 0.25, Number of Sources ≥ 10)^8^, and obtained 637, 519, 1,253, and 1,264 genes, respectively. Through retrieval related to miR-23a, microarray dataset GSE77298 of the GEO database revealed 4,150 significantly differentially expressed genes. The intersection of these results yielded two key downstream genes of miR-23a: PDE7A and MDM2 (Figure 3A). MDM2 has been shown to promote the occurrence of RA (Zhang et al., 2016). In addition, the starBase predicted the binding sites of miR-23a in human and mouse to MDM2 (Figure 3B). The dual luciferase reporter gene assay found that in HEK293T cells, miR-23a mimic inhibited the luciferase activity in the MDM2-Wt group, while had no effect on the luciferase activity in the MDM2-Mut group (Figure 3C). MDM2 was overexpressed in FLSs, which were treated by LPS, followed by determination of MDM2 expression. It was found that LPS treatment significantly increased MDM2 expression while further addition of miR-23a mimic resulted in decline of MDM2 expression. In FLSs treated with LPS in the presence of miR-23a mimic, the delivery of oe-MDM2 led to elevated MDM2 expression (Figure 3D). Subsequent cellular function evaluation and determination on secretion of inflammatory factors and expression of the NF-κB signaling pathway-related proteins showed that compared with the PBS group and the LPS + miR-23a mimic + oe-NC group, there was a significant increase in MDM2 expression, and an enhancement in the cell viability and secretion of inflammatory factors (IL-6, IL-1β, and TNF-α) and phosphorylation of p65 in the LPS group and the LPS + miR-23 a mimic + oe-MDM2 group; vs. the LPS + mimic-NC group, an inverse tendency was observed in the LPS + miR-23a mimic group (Figures 3D–G).

### MDM2 Ubiquitination Degrades SIRT6 and Promotes the NF-κB Signaling Pathway Activation in RA

**FIGURE 4:**
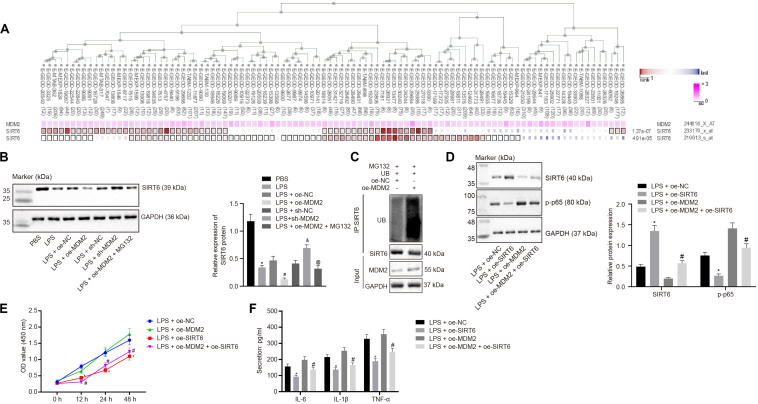
MDM2 ubiquitination degrades SIRT6 and promotes the activation of the NF-κB signaling pathway in RA. **(A)**. MEM analysis showed significant co-expression of MDM2 and SIRT6 (*p* = 1.37E-07). **(B)** Detection of SIRT6 expression normalized to GAPDH in FLSs by Western blot analysis. ^∗^*p* < 0.05 vs. the PBS group; #*p* < 0.05 vs. the LPS + oe-NC group; &*p* < 0.05 vs. the LPS + sh-NC group; @*p* < 0.05 vs. the LPS + oe-MDM2 group. **(C)** Immunoprecipitation (IP) assay showing the ubiquitination of SIRT6. **(D)** Detection of the expression of SIRT6 and phosphorylation of p65 normalized to GAPDH in FLSs by Western blot analysis. **(E)** Detection of viability of FLSs by CCK-8 assay. **(F)** Detection of the secretion of inflammatory factors in the culture medium of FLSs by ELISA. **(D–F)** ^∗^*p* < 0.05 vs. the LPS + oe-NC group; #*p* < 0.05 vs. the LPS + oe-MDM2 group. The measurement data were depicted as mean ± standard deviation. The data among multiple groups was analyzed using one-way ANOVA, followed by Tukey’s *post hoc* test. Cell viability at different time points was compared using two-way ANOVA, followed by Bonferroni *post hoc* test. Each experiment was run in triplicate.

Evidence has shown that MDM2 can inhibit the expression of SIRT6 through its E3 ubiquitinase function (Thirumurthi et al., 2014). To identify the interaction between MDM2 and SIRT6, co-expression analysis website MEM analysis^9^ was used for online prediction of target genes through co-expression. MDM2 and SIRT6 were predicted to be significantly co-expressed (*p* = 1.37E − 07) (Figure 4A). From the above-mentioned results, we found that miR-23a/MDM2 could affect the activation of NF-κB signaling pathway. In order to probe into whether the downstream signaling of MDM2 is related to SIRT6, we firstly tested the expression of SIRT6 in LPS-treated cell models. It was found that in comparison to the PBS group and the LPS + oe-NC group, SIRT6 expression reduced in the LPS group and the LPS + oe-MDM2 group; and compared with the LPS + sh-NC group, the expression of SIRT6 was increased in the LPS + sh-MDM2 group. After treatment with the proteasome inhibitor MG132, in contrast to the LPS + oe-MDM2 group, the decrease of SIRT6 protein in the LPS + oe-MDM2 + MG132 group was significantly reversed (Figure 4B), suggesting that MG132 could block the inhibitory effect of MDM2 on SIRT6. Meanwhile, we also found in the immunoprecipitation assay that overexpression of MDM2 promoted ubiquitination of SIRT6 protein (Figure 4C). The findings for whether SIRT6 could be involved in RA suggested that, relative to the LPS + oe-NC and the LPS + oe-MDM2 group, elevated SIRT6 and suppressed cell viability and secretion of inflammatory factors (IL-6, IL-1β, and TNF-α) and phosphorylation of p65 were found in the LPS + oe-SIRT6 and the LPS + oe-MDM2 + oe-SIRT6 group (Figures 4D–F). These results indicate that MDM2 ubiquitinates SIRT6 in RA, and MDM2 promotes activation of the NF-κB signaling pathway through SIRT6 to promote FLS viability and inflammation.

### LncRNA NEAT1 Shuttled by PBMC-Derived Exos Promotes FLS Viability and Inflammation Through MDM2/SIRT6

**FIGURE 5:**
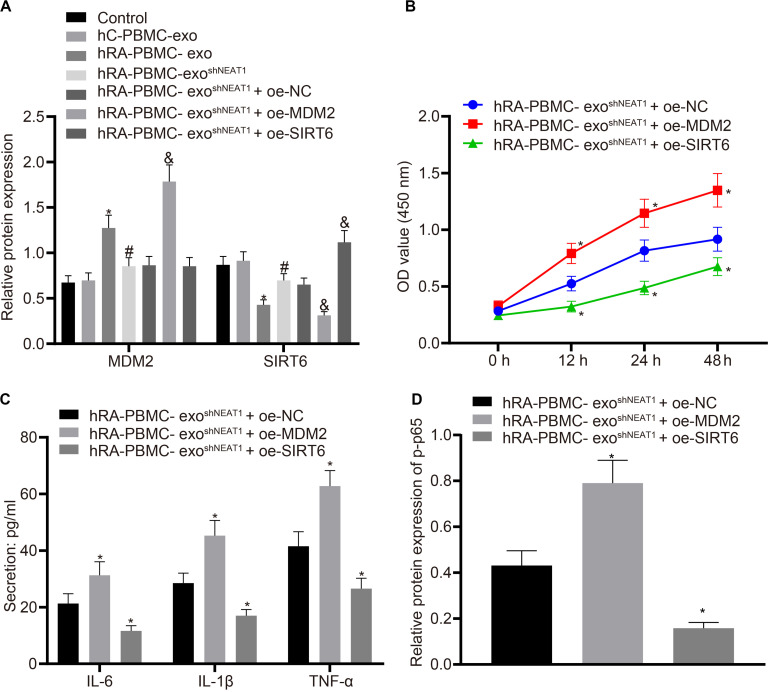
LncRNA NEAT1 shuttled by PBMC-derived exos promotes FLS viability and inflammation by modulating MDM2/SIRT6. The cells were assigned to the control (normal FLSs), hC-PBMC-exo (co-culture of exos secreted from normal human PBMCs with FLSs), hRA-PBMC-exo (co-culture of exos secreted from PBMCs of RA patients with FLSs), hRA-PBMC-exo^shNC^ (co-culture of exos secreted from PBMCs of RA patients that had been infected with sh-NC with FLSs), hRA-PBMC-exo ^shNEAT1^ (co-culture of exos secreted from PBMCs of RA patients that had been infected with shNEAT1 with FLSs), hRA-PBMC exo ^shNEAT1^ + oe-NC (co-culture of exos secreted from PBMCs of RA patients that had been infected with shNEAT1 with FLSs that had been transfected with oe-NC), hRA-PBMC-exo ^shNEAT1^ + oe-MDM2 (co-culture of exos secreted from PBMCs of RA patients that had been infected with shNEAT1 with FLSs that had been transfected with oe-MDM2) and hRA-PBMC-exo ^shNEAT1^ + oe-SIRT6 (co-culture of exos secreted from PBMCs of RA patients that had been infected with shNEAT1 with FLSs that had been transfected with oe-SIRT6) groups. **(A)** Detection of MDM2 and SIRT6 expression normalized to GAPDH in FLSs by Western blot analysis. ^∗^*p* < 0.05 vs. the hC-PBMC-exo group; #*p* < 0.05 vs. the hRA-PBMC-exo group; &*p* < 0.05 vs. the hRA-PBMC-exo ^shNEAT1^ + oe-NC group. **(B)** Detection of viability of FLSs by CCK-8 assay. **(C)** Detection of the secretion of inflammatory factors in the culture medium of FLSs by ELISA. **(D)** Detection of phosphorylation of p65 normalized to GAPDH in FLSs by Western blot analysis. **(B–D)** ^∗^*p* < 0.05 vs. the hRA-PBMC-exo ^shNEAT1^ + oe-NC group. The measurement data were depicted as mean ± standard deviation. The data among multiple groups was analyzed using one-way ANOVA, followed by Tukey’s *post hoc* test. Cell viability at different time points was compared using two-way ANOVA, followed by Bonferroni *post hoc* test. Each experiment was run in triplicate.

For the purpose of verifying whether the role of PBMC-derived exos in RA is related to MDM2/SIRT6, co-culture was performed to observe the effect of PBMC-derived exos on FLSs. Firstly, we tested the expression of MDM2 and SIRT6 in the co-culture system of PBMC-derived exos and FLSs. It was found that in comparison to the control group or hC-PBMC-exo group, the hRA-PBMC-exo group presented increased MDM2 expression and decreased SIRT6 expression; compared with the hRA-PBMC-exo group, the expression of MDM2 reduced, and the expression of SIRT6 elevated in the hRA-PBMC-exo ^*shNEAT1*^ group; vs. the hRA-PBMC-exo ^*shNEAT1*^ + oe-NC group, increased MDM2 expression and decreased SIRT6 expression were found in the hRA-PBMC-exo ^*shNEAT1*^ + oe-MDM2 group, while MDM2 expression had no significant change in the hRA-PBMC-exo ^*shNEAT1*^ + oe-SIRT6 group, and SIRT6 expression increased (Figure 5A). Additionally, we also found that relative to the hRA-PBMC-exo ^*shNEAT1*^ + oe-NC group, promoted viability and secretion of inflammatory factors (IL-6, IL-1β, and TNF-α) and phosphorylation of p65 were found in the hRA-PBMC-exo ^*shNEAT1*^ + oe-MDM2 group, while the hRA-PBMC-exo ^*shNEAT1*^ + oe-SIRT6 group presented an opposite trend (Figures 5B–D). These results suggest that lncRNA NEAT1 shuttled by PBMC-derived exos could promote FLS viability and inflammation through MDM2/SIRT6.

### Downregulation of LncRNA NEAT1 Inhibits FLS Viability and Inflammation in RA by Upregulating miR-23a

**FIGURE 6:**
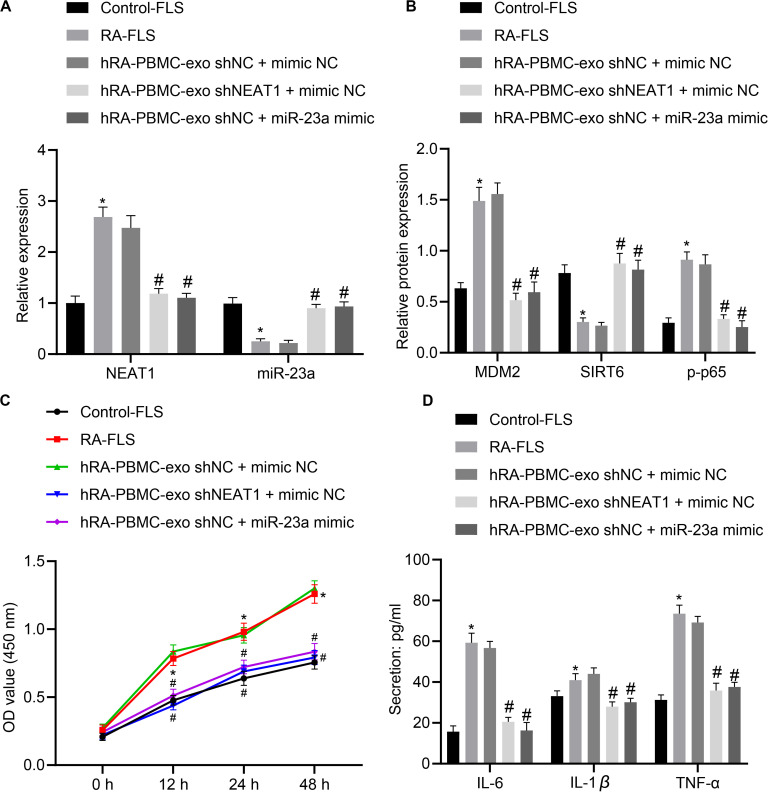
LncRNA NEAT1 knockdown shuttled by PBMC-derived exos curbs viability and inflammation of FLSs in RA. **(A)** RT-qPCR used to detect the expression of lncRNA NEAT1 and miR-23a in synovial tissues of mice in each group. **(B)** Western blot analysis used to detect the expression of MDM2, SIRT6 and phosphorylation of p65 normalized to GAPDH in the synovial tissues of mice in each group. **(C)** Detection of viability of FLSs by CCK-8 assay. **(D)** Detection of the secretion of inflammatory factors in the culture medium of FLSs by ELISA. *p < 0.05 vs. the control group; #*p* < 0.05 vs. the hC-PBMC-exo ^shNC^ + mimic NC group. The measurement data were depicted as mean ± standard deviation. The data among multiple groups was analyzed using one-way ANOVA, followed by Tukey’s *post hoc* test. *n* = 8. Each experiment was run in triplicate.

With the above finding determining the implication of lncRNA NEAT1 in viability and inflammation of FLSs through the NF-κB signaling pathway via the miR-23a/MDM2/SIRT6 axis, we then isolated and cultured FLSs from RA mice for further verification *in vivo*. According to quantification of lncRNA NEAT1, miR-23a/MDM2/SIRT6 and the extent of p65 phosphorylation, lncRNA NEAT1, MDM2 and the extent of p65 phosphorylation were significantly upregulated while miR-23a and SIRT6 expression was diminished in FLSs from RA mice (Figures 6A,B), accompanied by promoted cell viability and secretion of inflammatory factors (IL-6, IL-1β, and TNF-α) (Figures 6C,D). Next, RA-FLSs were co-cultured with exos derived from PBMCs in the presence of silenced lncRNA NEAT1 or miR-23a mimic. Results showed that exo ^*shNEAT1*^ or miR-23a mimic significantly downregulated expression of lncRNA NEAT1 and MDM2 and extent of p65 phosphorylation while upregulating miR-23a and SIRT6, accompanied by suppressed cell viability and secretion of inflammatory factors (IL-6, IL-1β, and TNF-α) (Figures 6A–D). Taken together, these findings suggest that downregulated lncRNA NEAT1 or upregulated miR-23a exerted inhibitory effect on FLS viability and inflammation in RA.

### Downregulation of LncRNA NEAT1 Shuttled by PBMC-Derived Exos Suppresses RA Deterioration of Mice

**FIGURE 7:**
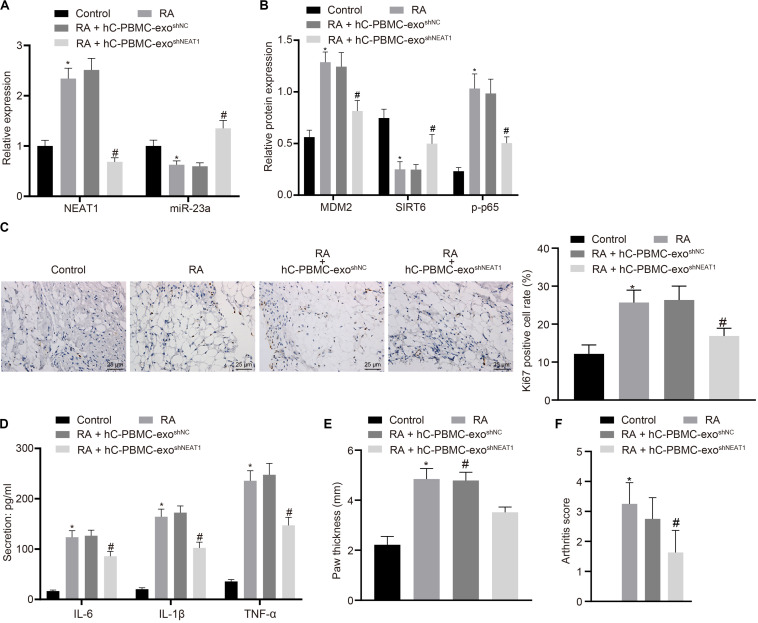
LncRNA NEAT1 knockdown shuttled by PBMC-derived exos restricts RA deterioration in mice. **(A)** RT-qPCR used to detect the expression of lncRNA NEAT1 and miR-23a in synovial tissues of mice in each group. **(B)** Western blot analysis used to detect the expression of MDM2, SIRT6 and phosphorylation of p65 normalized to GAPDH in the synovial tissues of mice in each group. **(C)** Detection of Ki67 expression in synovial tissues of mice in each group by Immunohistochemical staining (400 ×). **(D)** ELISA to detect the secretion of inflammatory factors in the serum of mice in each group. **(E)** Determination of the paw thickness of each group of mice. **(F)** Determination of the arthritis score of each group of mice. ^∗^*p* < 0.05 vs. the control group; #*p* < 0.05 vs. the hC-PBMC-exo ^shNC^ group. The measurement data were depicted as mean ± standard deviation. The data among multiple groups was analyzed using one-way ANOVA, followed by Tukey’s *post hoc* test. *n* = 8. Each experiment was run in triplicate.

In order to determine the role of lncRNA NEAT1 in RA *in vivo*, we injected PBMC-derived exos in mice on the 1st, 3rd, and 5th days (Wiklander et al., 2015) and then determined the expression of lncRNA NEAT1 and miR-23a/MDM2/SIRT6, and phosphorylation of p65 in mouse synovial tissues. The results indicated that the expressions of lncRNA NEAT1, MDM2, and phosphorylation of p65 increased in the synovial tissues of RA mice, while the expressions of miR-23a and SIRT6 reduced. Compared with the hC-PBMC-exo^shNC^ group, the expression of lncRNA NEAT1, MDM2, and phosphorylation of p65 reduced in the hC-PBMC-exo ^*shNEAT1*^ group, while the expression of miR-23a and SIRT6 increased (Figures 7A,B). As one of the most widely used indicators of proliferative cells, Ki67, localized in the nucleus, is a nuclear antigen present in proliferative cells and its immunoreaction is closely related to cell cycle (Yang et al., 2018). Meanwhile, cell proliferation was under observation to evaluate the RA deterioration of mice. Determination of Ki67 expression by immunohistochemistry in synovial tissues revealed that Ki67 expression elevated in the synovial tissues of RA mice. In comparison to the RA + hC-PBMC-exo ^*shNC*^ group, Ki67 expression reduced in the RA + hC-PBMC-exo^shNEAT1^ group (Figure 7C). The secretion of serum inflammatory factors (IL-6, IL-1β, and TNF-α) in mice were also measured, and the results showed that the expression of IL-6, IL-1β, and TNF-α increased as a validation of RA model. Contrasted to the RA + hC-PBMC-exo^shNC^ group, the expression of IL-6, IL-1β, and TNF-α reduced in the RA + hC-PBMC-exo ^*shNEAT1*^ group (Figure 7D). When the RA mouse models were established for 5 weeks, the paw thickness and arthritis score of the mice in each group were measured, and the results revealed that the paw thickness and arthritis score enhanced in RA mice. Compared with the RA + hC-PBMC-exo ^*shNC*^ group, the paw thickness and arthritis score declined in the RA + hC-PBMC-exo ^*shNEAT1*^ group (Figures 7E,F). These results suggest that downregulation of lncRNA NEAT1 in PBMC-derived exos could inhibit RA deterioration in mice.

### LncRNA NEAT1 Loaded in Exos Derived From PBMCs of RA Patients Aggravates RA in Mice

**FIGURE 8:**
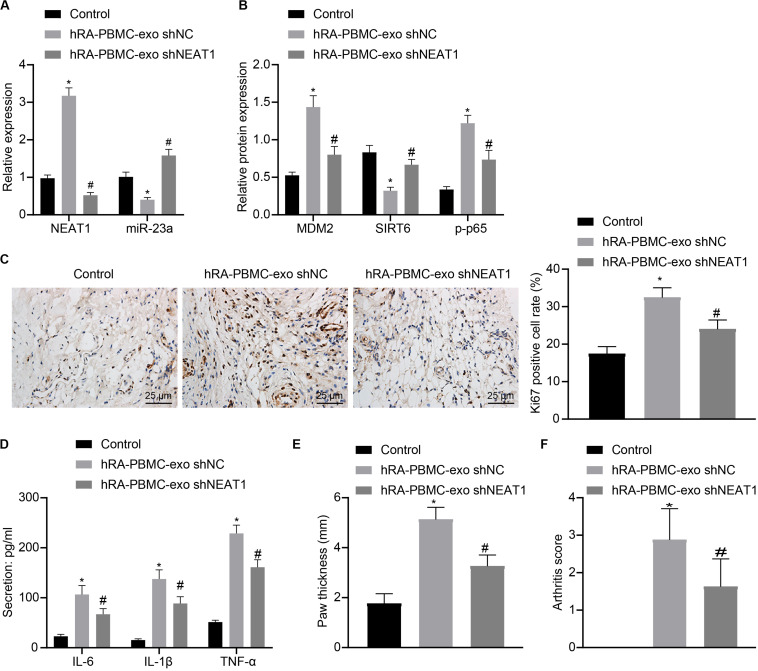
LncRNA NEAT1 shuttled by PBMC-derived exos from RA patients facilitates RA deterioration in mice. **(A)** RT-qPCR used to detect the expression of lncRNA NEAT1 and miR-23a in synovial tissues of mice in each group. **(B)** Western blot analysis used to detect the expression of MDM2, SIRT6 and phosphorylation of p65 normalized to GAPDH in the synovial tissues of mice in each group. **(C)** Detection of Ki67 expression in synovial tissues of mice in each group by Immunohistochemical staining (400 ×). **(D)** ELISA to detect the secretion of inflammatory factors in the serum of mice in each group. **(E)** Determination of the paw thickness of each group of mice. **(F)** Determination of the arthritis score of each group of mice. *p < 0.05 vs. the control group; #*p* < 0.05 vs. the hC-PBMC-exo ^shNC^ group. The measurement data were depicted as mean ± standard deviation. The data among multiple groups was analyzed using one-way ANOVA, followed by Tukey’s *post hoc* test. *n* = 8. Each experiment was run in triplicate.

To further verify the action of lncRNA NEAT1 in RA, control mice were used and injected with PBMC-derived exos from RA patients, which induced higher levels of lncRNA NEAT1, MDM2 and p-p65 in synovial tissues where miR-23a and SIRT6 levels were reduced. When lncRNA NEAT1 was silenced, levels of lncRNA NEAT1, MDM2 and p-p65 were lowered while miR-23a and SIRT6 levels were elevated (Figures 8A,B). Ki67 expression by immunohistochemistry and secretion of inflammatory factors (IL-6, IL-1β, and TNF-α) by ELISA revealed that Ki67 level in mouse synovial tissues and secretion of inflammatory factors (IL-6, IL-1β, and TNF-α) in mouse serum were both potentiated in the presence of PBMC-derived exos from RA patients while further delivery of shNEAT1 significantly reversed the results (Figures 8C,D). Furthermore, paw thickness and arthritis score were higher after injection of PBMC-derived exos from RA patients for 5 weeks yet lower in response to shNEAT1 (Figures 8E,F). To conclude, these results suggest that lncRNA NEAT1 loaded in PBMC-derived exos from RA patients gave rise to similar symptoms of RA, which were alleviated when lncRNA NEAT1 was silenced, indicating that lncRNA NEAT1 secreted by PBMC-derived exos from RA patients contributed to the occurrence of RA.

## Discussion

Notably, emerging evidence has suggested that lncRNAs are vital modulators of pathologic and physiologic processes, and lncRNAs are significant regulators of the inflammatory response (Pearson and Jones, 2016). Prior evidence has indicated that lncRNA NEAT1 plays a role in the modulation of multiple genes and pathways, thereby inducing inflammation disorder and infection, as well as exaggerating inflammatory response and infection (Huang et al., 2018). However, its role in RA has not been fully explored. Thus, investigations on the inflammation in RA may lead to a better understanding of RA pathogenesis and aid in the recognition of novel therapeutic targets. Altogether, this study suggests that lncRNA NEAT1 shuttled by PBMCs-derived exos promotes the development of RA by regulating miR-23a/MDM2/SIRT6 axis.

We found that lncRNA NEAT1 was upregulated in RA and PBMC-derived exos resulted in RA by delivering lncRNA NEAT1. Additionally, the *in vivo* experiments suggested that downregulation of lncRNA NEAT1 shuttled by PBMC-derived exos impeded RA deterioration of mice. It has been demonstrated that lncRNA NEAT1 restricted cell growth, enhanced the apoptotic rate and the in?ammatory cytokines released in OA chondrocytes (Wang et al., 2019). Similar to our results, lncRNA NEAT1 expression was found to be upregulated in OA tissues, and the concentrations of inflammatory factors were declined, cell viability of synoviocyte was inhibited by lncRNA NEAT1 knockdown (Wang et al., 2017). The results of another study have shown that lncRNA NEAT1 is upregulated in PBMCs of patients with RA, and the therapeutic effective of depleted lncRNA NEAT1 on alleviating the inflammatory degree was confirmed in arthritis mouse models (Shui et al., 2019). Moreover, evidence has shown that lncRNA NEAT1 is highly expressed in PBMC-derived exos from patients with RA (Song et al., 2015).

In addition, we also found that overexpression of miR-23a inhibited the promotion of FLS viability and inflammation by PBMC-derived exos in patients with RA. It is reported that deficiency of miR-23a induced cytoskeletal rearrangement, and promoted migration, invasion as well as expression of pro-inflammatory cytokines in PsA synovial fibroblasts (Wade et al., 2019). Meanwhile, it has been revealed that miR-23a-3p is upregulated in OA cartilage, and upregulated miR-23a-3p inhibited chondrocyte extracellular matrix synthesis and contributed to the progression of OA (Kang et al., 2016). In line with our finding, miR-23a was found to be poorly expressed in articular cartilage tissues from patients with RA. miR-23a suppressed activation of inflammatory cytokine-induced NF-κB and expression of some proinflammatory mediators in articular chondrocytes (Hu et al., 2017). In this current study, we also found that there was a binding site between miR-23a and MDM2, and miR-23a inhibited inflammation in RA by suppressing MDM2. As previously described, suppression of MDM2 exhibits anti-inflammatory activity, which might be a novel therapeutic target for RA (Zhang et al., 2016). However, the relationship between miR-23a and MDM2 needs further confirmation. In addition, we found that there was a bindings site between lncRNA NEAT1 and miR-23a. LncRNAs have been confirmed to impact gene expression via multiple mechanisms, including chromatin remodeling, mRNA stabilization, competing endogenous RNAs, as well as recruitment of scaffolding proteins (Sun et al., 2019). This information helps to elucidate their vital roles in disease development. Zhang et al. have found that the correlation between miR-23a-3p and lncRNA NEAT1 (Zhao et al., 2019), which is in accordance with our result.

Furthermore, another finding of this study suggested that MDM2 ubiquitination degraded SIRT6 and promoted the NF-κB signaling pathway activation in RA. As a member of the sirtuin family, SIRT6 has received much attention due to its functions in DNA repair, genomic stability and restriction of tumor formation and the NF-κB signaling (Beauharnois et al., 2013). Meanwhile, SIRT6 has been demonstrated to attenuate the NF-κB signaling and also, decrease NF-κB-dependent pro-inflammatory genes expression (Kawahara et al., 2009). Another study has proposed that SIRT6 is able to attenuate the NF-κB signaling through H3K9 deacetylation at chromatin (Kawahara et al., 2009). Furthermore, it is revealed that overexpressed MDM2 declined SIRT6 abundance in cells, but decreasing the endogenous MDM2 enhanced SIRT6 abundance, implying that MDM2 degrades SIRT6 in a dependent manner (Thirumurthi et al., 2014).

Collectively, this study indicates that downregulation of lncRNA NEAT1 shuttled by PBMC-derived exos suppresses RA deterioration *via* the miR-23a/MDM2/SIRT6 axis. This study has provided lncRNA NEAT1, miR-23a and MDM2 as candidate diagnostic biomarkers or potential therapeutic targets for RA and will broaden our understanding of the pathogenesis underlying RA.

## Data Availability Statement

The raw data supporting the conclusions of this article will be made available by the authors, without undue reservation, to any qualified researcher.

## Ethics Statement

The studies involving human participants were reviewed and approved by the Affiliated Hospital of Yangzhou University. The patients/participants provided their written informed consent to participate in this study. The animal study was reviewed and approved by Affiliated Hospital of Yangzhou University.

## Author Contributions

YR and YF wrote the main manuscript. WT and DL ran the analysis. YP and XW conceived and designed the experiments. CZ, GL, and YR designed the study. All authors reviewed the manuscript.

## Conflict of Interest

The authors declare that the research was conducted in the absence of any commercial or financial relationships that could be construed as a potential conflict of interest.

## Footnotes

1 http://starbase.sysu.edu.cn/

2 http://www.bioconductor.org/packages/release/bioc/html/limma.html

3 https://www.ncbi.nlm.nih.gov/geo/query/acc.cgi

4 https://cm.jefferson.edu/rna22/

5 http://starbase.sysu.edu.cn/

6 http://mirwalk.umm.uni-heidelberg.de/

7 http://diana.imis.athena-innovation.gr/DianaTools

8 http://ophid.utoronto.ca/mirDIP/

9 https://biit.cs.ut.ee/mem/

## Abbreviations

ANOVA: analysis of variance
CCK-8: cell counting kit-8
ELISA: enzyme-linked immunosorbent assay
Exo: exosome
FLSs: fibroblast-like synoviocytes
GAPDH: glyceraldehyde phosphate dehydrogenase
GEO: Gene Expression Omnibus
IL: interleukin
LncRNA: long non-coding RNA
MDM2: murine double minute-2
miR-23a: microRNA-23a
Mut: mutant type
NF- κ B: nuclear factor kappa B
PBMC: peripheral blood monouclear cell
PsA: psoriatic arthritis
RA: rheumatoid arthritis
RT-qPCR: Reverse transcription quantitative polymerase chain reaction
SDS-PAGE: sodium dodecyl sulfate polyacrylamide gel electrophoresis
SIRT6: Sirtuin 6
TNF: tumor necrosis factor
UB: ubiquitin
Wt: wild type.
